# Differential Effects of Group Intervention on Psychological Subhealth in University Students: Evidence from Core Predicaments

**DOI:** 10.3390/bs15121738

**Published:** 2025-12-16

**Authors:** Anxin Li, Yanan Meng

**Affiliations:** Department of Law and Political Science, North China Electric Power University (Baoding), Baoding 071003, China; anxinli@ncepu.edu.cn

**Keywords:** university students, psychological subhealth, group intervention, differential effects, core predicaments, SCL-90

## Abstract

Psychological subhealth among university students is a growing concern affecting their development, with insufficient attention to differential intervention effects for students facing distinct core predicaments. This exploratory study investigated the overall and differential effects of a group intervention integrating cognitive behavioral and social support theories on university students with psychological subhealth experiencing trauma, academic pressure, or family dysfunction. After defining these core predicament groups through questionnaire screening, twenty students with psychological subhealth were recruited from each of the three groups (total *N* = 60) and subsequently randomly assigned to either an 8-week group intervention or a control group. The Symptom Checklist-90 (SCL-90) was used for pre- and post-test assessments. Results showed the intervention significantly improved students’ overall psychological health and depressive symptoms. Differential effects were evident: the trauma group markedly improved in interpersonal sensitivity and anxiety; the academic pressure group showed pronounced improvement in obsessive-compulsive and anxiety symptoms; and the family dysfunction group benefited most regarding interpersonal sensitivity and hostility. This study suggests the intervention’s effectiveness and its link to students’ core predicament types, indicating a need for differentiated strategies based on precise assessment. These findings provide practical implications for precision-oriented mental health services in higher education.

## 1. Introduction

The mental health of university students is critical not only for their personal academic and developmental outcomes but also for the broader well-being of society. The World Health Organization’s World Mental Health Report underscores the critical importance of mental health for every individual and region (World Health Organization, 2022), while Bantjes et al. (2022) have highlighted the commonality of mental disorders and suicidal ideation among university students globally. In recent years, “psychological subhealth” has become increasingly prominent. Unlike specific “subclinical disorders” (which imply a milder form of a distinct pathology) or general “psychological distress,” psychological subhealth refers to a borderline state between health and illness widely recognized in East Asian contexts. It is characterized by significant psychological distress, emotional fluctuations, and social maladjustment (e.g., chronic fatigue, loss of interest, or interpersonal sensitivity) that persist but do not meet the diagnostic criteria for severe mental disorders (Zhang et al., 2022). It has emerged as a widely recognized public health issue within the global higher education sector (Wang et al., 2024). Numerous studies have identified multiple risk factors associated with psychological subhealth in university students, which may act independently or concertedly. Key risk factors include persistent academic pressure, closely linked to emotional health problems such as anxiety and depression (Mofatteh, 2021). Furthermore, interpersonal relationship difficulties, pre-existing traumatic experiences, and complex family environments are also considered significant contributing factors (Lin et al., 2024; Griffith et al., 2020). Challenges in adapting to new university environments, such as changes in social and academic expectations, also elevate the risk of psychological subhealth (Mofatteh, 2021). Without timely and effective intervention, these issues can escalate, severely impacting academic achievement, social functioning, and long-term development (Barbayannis et al., 2022; Mofatteh, 2021). Against this backdrop, exploring effective, universally applicable, yet targeted psychological intervention strategies to enhance university students’ mental health has become a crucial task for ensuring the quality of higher education, promoting holistic individual development, and maintaining social stability.

To address psychological subhealth among university students, academia and practitioners have explored and implemented various intervention pathways, including individual counseling, mental health education courses, and online intervention platforms (Cerolini et al., 2023; Hargreaves et al., 2025; Ferrari et al., 2022). Among these, group work has demonstrated unique application potential due to its dual supportive and task-oriented nature, fostering an accepting atmosphere and providing emotional and informational support while guiding members to learn coping skills and adjust cognitive patterns (Toseland & Rivas, 2014; Worsley et al., 2022). Cognitive Behavioral Theory (CBT) and Social Support Theory are well-established theoretical frameworks guiding mental health interventions. CBT emphasizes improving emotional and behavioral problems by identifying and modifying non-adaptive cognitions, combined with behavioral skills training (A. T. Beck et al., 1979; J. S. Beck, 2020). Conversely, Social Support Theory focuses on buffering stress and promoting adaptation by enhancing individuals’ social connections and perceived support resources (Cohen & Wills, 1985; Cobb, 1976). Integrating these two theories into group interventions offers a promising approach to synergistically promoting university students’ mental health from both internal cognitive adjustment and external environmental support perspectives.

However, despite progress in existing intervention research, several aspects warrant further investigation. Firstly, many intervention programs tend to adopt a “one-size-fits-all” standardized approach in their design and implementation, often inadequately considering the significant heterogeneity within the university student population. Individuals may differ considerably in their manifestations of psychological subhealth, intrinsic needs, and response patterns to interventions due to unique developmental experiences, personality traits, and specific stressors they face (i.e., “core predicaments”). This oversight of individual differences may limit the universality and optimal effectiveness of interventions. Secondly, although the potential of group interventions is recognized, empirical research specifically examining the differential effects of interventions for university student groups facing distinct core predicaments remains relatively scarce, particularly studies exploring which intervention elements are most effective for specific predicament groups within particular cultural contexts (e.g., China). Therefore, a deeper understanding and systematic comparison of the specific benefit patterns of integrated group interventions among university students with different core predicaments are of critical theoretical and practical significance for transitioning mental health services from universal coverage to precision support.

Based on the review of relevant literature and theoretical considerations outlined above, this study employed a pre-test post-test control group design to examine the effects of a group intervention integrating CBT and the Social Support Theory. Rather than a generic application, we sought to investigate the intervention’s differential impacts on university students with psychological subhealth facing three distinct core predicaments: traumatic experiences, significant academic pressure, and family dysfunction. We hypothesized that while the intervention would improve overall psychological health (e.g., SCL-90 total scores) for all participants, specific symptom dimensions would show differential improvements corresponding to the unique nature of each group’s core predicament.

## 2. Materials and Methods

### 2.1. Study Design

This study employed a pretest-posttest control group design to investigate the intervention effects and differential characteristics of a group intervention, integrating cognitive behavioral theory and social support theory, on university students with psychological subhealth facing different core predicaments. The research procedure primarily involved defining a sample of university students with psychological subhealth through questionnaire screening, further classifying them based on core predicament types, recruiting participants from each category and randomly assigning them to either an intervention group or a waitlist control group, collecting baseline data (pre-test T1), implementing an 8-week group intervention (the control group did not receive this intervention), and collecting post-intervention assessment data (post-test T2).

### 2.2. Participants, Sampling Procedure, and Measurement Tools

#### 2.2.1. Population and Initial Screening

The participants in this study were full-time undergraduate students from several universities in the Beijing-Tianjin-Hebei region of China. To identify potential participants meeting the criteria for psychological subhealth, an initial large-scale online screening was conducted using the Symptom Checklist-90 (SCL-90). The SCL-90 is a widely used psychological health assessment tool comprising 90 items designed to evaluate the severity of various psychological symptoms experienced by an individual over the past week, with items rated on a 5-point Likert scale (from “Not at all” to “Extremely”). The scale covers nine factor dimensions: somatization, obsessive-compulsive, interpersonal sensitivity, depression, anxiety, hostility, phobic anxiety, paranoid ideation, and psychoticism, and a total score can be calculated. In this study, the SCL-90 total score was primarily used as the initial criterion for defining psychological subhealth, with changes in both the total score and factor scores being a focus in the subsequent intervention effect evaluation. The SCL-90 has demonstrated good reliability and validity among university student populations in both Chinese and international university student populations (Yu et al., 2019). Through this initial screening, an effective sample of *N* = 5428 university students with SCL-90 total scores between 160 and 225, meeting the study’s preliminary criteria for psychological subhealth, was obtained.

#### 2.2.2. Classification of Core Predicaments

From the aforementioned sample of 5428 university students preliminarily identified with psychological subhealth, this study utilized a self-developed “University Students’ Core Predicament Screening and Classification Questionnaire” for further detailed classification based on their core predicaments. The development of this questionnaire was informed by the Adolescent Self-Rating Life Events Checklist (ASLEC) (Liu et al., 1997) and tailored to the specific objectives of this research. It aimed to assess participants’ distress levels across three core predicament dimensions: (1) trauma-related experiences, (2) significant academic pressure, and (3) family dysfunction. All items in the questionnaire were rated on a 5-point Likert scale, and cumulative scores were calculated for each participant on each dimension.

To ensure the purity of single-predicament characteristics and inter-group heterogeneity for subsequent intervention grouping, this study employed a strict dual-threshold, mutually exclusive allocation principle based on the “University Students’ Core Predicament Screening and Classification Questionnaire” data from the 5428 students with psychological subhealth:

(1) Threshold Determination: A percentile analysis was conducted on the score distribution for each core predicament dimension. The 80th percentile (P80) of the score distribution for a given dimension was set as the high-stringency inclusion threshold (Upper Threshold, UT); participants scoring ≥UT were considered to have “significant distress” in that dimension. Concurrently, the 20th percentile (P20) of the score distributions for the other two non-target dimensions were set as high-stringency exclusion thresholds (Lower Threshold, LT); participants scoring ≤LT on these dimensions were considered to have “no significant distress” in those respective dimensions.

(2) Group Definition Criteria: An individual was assigned to a specific core predicament group if, and only if, their score was ≥UT on the target dimension and simultaneously ≤LT on both of the other two non-target dimensions.

This questionnaire served as a key tool in this study for differentiating participants into distinct core predicament subgroups, and its preliminary internal consistency reliability test yielded a Cronbach’s α coefficient of 0.916. This rigorous screening strategy, based on dual-threshold and mutually exclusive allocation, was designed to maximize the internal homogeneity of each core predicament subgroup and the significant differences between groups. This approach aimed to establish a solid foundation for clearly examining the targeted effects of the group intervention on specific types of distressed populations, although it also implied a highly selective screening process from a relatively large initial sample to a smaller number of participants meeting the stringent criteria for the final intervention study.

#### 2.2.3. Final Participant Ascertainment and Grouping

Following the definition of psychological subhealth status via the SCL-90 and the identification of a potential participant pool facing a single core predicament (i.e., traumatic experiences, significant academic pressure, or family dysfunction) through the “University Students’ Core Predicament Screening and Classification Questionnaire,” researchers recruited final participants for the intervention study from this pool.

The final inclusion criteria were (1) an SCL-90 total score between 160 and 225, meeting the criteria for psychological subhealth; (2) age between 18 and 25 years; (3) clear assignment to one of the three core predicament groups based on the aforementioned questionnaire assessments, strictly adhering to the single-predicament characteristic criteria; and (4) voluntary participation in the study with signed written informed consent.

The final exclusion criteria included (1) not meeting the SCL-90 psychological subhealth criteria or the single core predicament criteria (e.g., exhibiting multiple or mixed predicaments on the “University Students’ Core Predicament Screening and Classification Questionnaire”); (2) currently receiving other systematic psychological counseling, psychotherapy, or psychiatric medication; (3) presence of severe suicide risk; and (4) inability to guarantee full participation throughout the study.

Ultimately, 20 university students who met the criteria were recruited from each core predicament group, totaling 60 participants (*N* = 60). Within their respective predicament groups, participants were randomly assigned, using a random number table method, to either the intervention group (*n* = 10 per predicament subgroup, 30 in total) or the waitlist control group (*n* = 10 per predicament subgroup, 30 in total). The study received ethical approval from the ethics committee of the corresponding author’s department. All participants provided written informed consent before their involvement in the study.

### 2.3. Intervention Protocol

#### 2.3.1. Intervention Group

The three intervention subgroups (traumatic experience intervention group, significant academic pressure intervention group, and family dysfunction intervention group, each *n* = 10) all received an 8-week standardized, structured social work group intervention, conducted once per week for 90 min. All groups were led by professional social workers/psychological counselors who had received uniform training on this study’s intervention protocol, strictly adhering to a consistent theoretical framework (integrating cognitive behavioral theory and social support theory) and a pre-designed core activity structure.

The intervention protocol integrated strategies and techniques from cognitive behavioral theory, such as psychoeducation, emotion identification and regulation techniques (e.g., mindfulness breathing, relaxation training), cognitive assessment and restructuring methods (e.g., identifying automatic thoughts, challenging irrational beliefs, thought records), expansion of stress-coping strategy repertoires, and interpersonal communication skills training (e.g., DESC communication scripts, nonviolent communication). Concurrently, the intervention emphasized the application of social support theory, utilizing group dynamics to foster a safe, accepting, and trusting atmosphere, thereby promoting emotional expression, experience sharing, mutual support, and empowerment among members.

The eight group sessions were structured around the following common thematic sequence:

- Week 1: Group formation and goal orientation.
- Week 2: Understanding core predicaments and psychoeducation on mental health.
- Week 3: Learning and practicing emotion regulation skills.
- Week 4: Identifying and assessing cognitive patterns.
- Week 5: Practicing cognitive restructuring techniques.
- Week 6: Expanding and selecting coping strategies.
- Week 7: Interpersonal communication skills training.
- Week 8: Integration, maintenance, and future planning.

To ensure the ecological validity and individual relevance of the intervention content for participants in each subgroup, a key aspect of the intervention, following the standardized teaching of core skills (e.g., universal concept instruction and skill demonstrations, which remained consistent across all groups), was guiding members to flexibly apply the learned general principles and techniques to their respective core predicament areas. Specifically, although the instruction of core concepts and skill demonstrations (e.g., mindfulness breathing, thought records, nonviolent communication sentence structures) was uniform across all groups, the focus of group discussions, case analyses, role-playing, and homework assignments naturally gravitated towards the primary issues of each group. For instance:

- During the emotion regulation module (Week 3), all members learned the same relaxation techniques, but the situational context for practice was tailored to managing specific emotional responses triggered by their respective core predicaments (e.g., trauma triggers, academic evaluation situations, or family conflicts).
- In cognitive restructuring (Week 5), cognitive error identification and disputation techniques were applied uniformly, but the automatic thought samples analyzed were primarily derived from members’ typical negative cognitions within their respective core predicaments (e.g., traumatic experiences, academic achievements, or family interactions).
- In interpersonal communication training (Week 7), a consistent communication model (e.g., DESC script) was taught, but the simulated scenarios for practice focused, respectively, on issues related to their respective core predicaments, such as setting safety boundaries related to trauma, seeking help or collaborating on academic matters, or addressing sensitive dialogs within the family.

This application-level adaptability aimed to enhance the congruence between the intervention measures and participants’ personal experiences, thereby intending to improve treatment engagement and skill generalization. Crucially, this strategy strictly maintained the fidelity of the core intervention protocol, ensuring that all experimental groups received equivalent theoretical input, technical training, and structured processes, without introducing additional therapeutic components.

#### 2.3.2. Control Group

The three control subgroups (traumatic experience control group, significant academic pressure control group, and family dysfunction control group, each *n* = 10) served as a waitlist control group. During the study period, the control groups did not receive the specific group intervention designed for this research and maintained their routine academic and daily life activities. Upon completion of the study, participants in the control group were offered the opportunity to participate in the same intervention program if they wished.

### 2.4. Statistical Analysis

Data were analyzed using SPSS 26.0. Baseline homogeneity was assessed using independent samples *t*-tests and chi-square tests. To rigorously evaluate the intervention’s differential effects and mitigate Type I errors, this study adopted a strict dual-criterion approach for interpreting improvements: a symptom dimension was considered effectively improved only if it demonstrated both (1) significant within-group improvement (via paired samples *t*-tests) and (2) a significant net intervention effect compared to the control group (via independent samples *t*-tests on change scores). Given the exploratory nature of this study and the subgroup sample size (*n* = 10), we prioritized the analysis of “change scores” over Repeated Measures ANOVA. Testing the difference in change scores provides a direct and robust assessment of the net intervention effect (conceptually equivalent to the Group × Time interaction) while avoiding potential power reduction associated with complex interaction models in smaller samples. Effect sizes were assessed using Cohen’s d (0.2 = small, 0.5 = medium, 0.8 = large). Finally, to control the False Discovery Rate (FDR) across the multiple comparisons (k = 60), the Benjamini–Hochberg (B-H) procedure was applied (FDR = 0.05), with statistical significance determined by the B-H adjusted thresholds.

## 3. Results

### 3.1. Baseline Level Comparison and Homogeneity Test of Groups

To examine the effectiveness of random assignment, this study compared the intervention and control groups within each core predicament subgroup (traumatic experience group, significant academic pressure group, and family dysfunction group) on demographic variables (e.g., age, gender, academic year distribution) and SCL-90 pre-test scores (total score and 9 factor scores) at baseline (T1). The results of independent samples *t*-tests (for continuous variables) and chi-square tests (for categorical variables) indicated no statistically significant differences (all *p* > 0.05) between the intervention and control groups within each core predicament subgroup on all measured demographic variables (age, gender, academic year) and SCL-90 pre-test scores. This suggests that the random assignment was effective, and the participant groups exhibited good homogeneity and comparability at the initial state, providing a reliable foundation for subsequent evaluation of intervention effects.

### 3.2. Overall Effects and Differential Characteristics

Given the multiple statistical inferences (k = 60 tests), the Benjamini-Hochberg (B-H) procedure was employed to control the False Discovery Rate (FDR) at α = 0.05. The adjusted significance threshold was determined using the formula:

p(i) ≤ (i/k) × α, where k = 60, α = 0.05(1)

Through calculation, the B-H adjusted significance threshold for this study was q = 0.026. Therefore, in the subsequent analysis of intervention effects, results with an original *p*-value less than or equal to 0.026 were considered statistically significant. For effect sizes, Cohen’s *d* ≥ 0.5 was considered a medium effect, and *d* ≥ 0.8 was considered a large effect.

#### 3.2.1. Traumatic Experience Group

(1) Within-group improvement: As shown in Table 1, after the intervention (T2), the traumatic experience intervention group showed significantly lower scores compared to pre-intervention (T1) on the SCL-90 total score and on eight dimensions: obsessive-compulsive, interpersonal sensitivity, depression, anxiety, phobic anxiety, paranoid ideation, and psychoticism (all *p* ≤ 0.026). Effect sizes for these significant improvements were large. Improvements on the somatization and hostility dimensions did not reach statistical significance after B-H correction (*p* > 0.026). Regarding the stability of scores, the zero-order correlations (r) between pre- and post-test scores varied across dimensions. While somatization showed a negative correlation (−0.364), the majority of dimensions (7 out of 10) exhibited strong positive correlations (r > 0.6), with the total score correlation at r = 0.877, reflecting a generally consistent pattern of symptom change.

(2) Net intervention effect: As indicated in Table 2, compared to the traumatic experience control group, the traumatic experience intervention group demonstrated significantly greater improvement (change scores) on the SCL-90 total score and on six dimensions: interpersonal sensitivity, depression, anxiety, phobic anxiety, and psychoticism (all *p* ≤ 0.026). Effect sizes indicated large or very large effects. The net intervention effects for the somatization, obsessive-compulsive, hostility, and paranoid ideation dimensions did not reach statistical significance after B-H correction (*p* > 0.026).

(3) Summary (Traumatic Experience Group): Comprehensive analysis indicated that the group intervention produced significant and practically meaningful improvements in overall psychological health and on specific symptom dimensions—including interpersonal sensitivity, depression, anxiety, phobic anxiety, and psychoticism—for university students with psychological subhealth who had traumatic experiences (meeting the criteria of both significant within-group improvement with *p* ≤ 0.026 and notable effect size and significant net intervention effect with *p* ≤ 0.026 and notable effect size).

**Table 1:** The *t*-test results of paired samples before and after the “traumatic experience group” experimental group.

SCL-90 Dimension	M ± SD		t	*p*	Cohen’s *d*
	Experimental Group Pre-Test	Experimental Group Post-Test			
Somatization	1.591 ± 0.253	1.258 ± 0.247	2.551	0.031	0.807
Obsessive-Compulsive	2.520 ± 0.424	2.380 ± 0.368	3.096	0.013	0.979
Interpersonal Sensitivity	2.512 ± 0.493	1.805 ± 0.484	6.932	0.000	2.192
Depression	2.423 ± 0.747	1.345 ± 0.216	5.465	0.000	1.728
Anxiety	2.190 ± 0.633	1.260 ± 0.310	6.268	0.000	1.982
Hostility	2.066 ± 0.465	2.111 ± 0.385	−0.444	0.667	0.141
Phobic Anxiety	2.219 ± 0.497	1.117 ± 0.177	9.031	0.000	2.856
Paranoid Ideation	2.133 ± 0.391	1.927 ± 0.346	3.026	0.014	0.957
Psychoticism	1.930 ± 0.521	1.090 ± 0.110	5.036	0.001	1.593
Total Score	190.9 ± 26.967	115.8 ± 15.288	15.412	0.000	4.874

Note: M = Mean; SD = Standard Deviation. Statistical significance was determined at *p* ≤ 0.026 after Benjamini–Hochberg correction for multiple comparisons (False Discovery Rate controlled at α = 0.05). Effect sizes (Cohen’s *d*) ≥ 0.5 are considered medium, and ≥0.8 are considered large. This significance criterion and these effect size interpretations apply to all subsequent tables presenting inferential statistics (Table 2, Table 3, Table 4, Table 5 and Table 6) unless otherwise specified.

**Table 2:** The independent sample *t*-test results of the change values between the experimental group and the control group in the “traumatic experience group”.

SCL-90 Dimension	M ± SD		t	*p*	Cohen’s *d*
	Experimental Group Change Scores	Control Group Change Scores			
Somatization	−0.333 ± 0.413	−0.123 ± 0.345	−1.235	0.233	0.552
Obsessive-Compulsive	−0.140 ± 0.143	−0.090 ± 0.597	−0.258	0.800	0.115
Interpersonal Sensitivity	−0.707 ± 0.323	−0.060 ± 0.155	−5.719	0.000	2.558
Depression	−1.078 ± 0.624	−0.247 ± 0.511	−3.258	0.004	1.457
Anxiety	−0.930 ± 0.469	0.030 ± 0.562	−4.147	0.001	1.855
Hostility	0.045 ± 0.320	−0.240 ± 0.691	1.184	0.252	0.529
Phobic Anxiety	−1.102 ± 0.386	−0.321 ± 0.719	−3.029	0.007	1.355
Paranoid Ideation	−0.206 ± 0.215	−0.270 ± 0.528	0.355	0.727	0.159
Psychoticism	−0.840 ± 0.527	−0.250 ± 0.422	−2.761	0.013	1.235
Total Score	−75.1 ± 15.409	−14.1 ± 19.116	−7.856	0.000	3.513

#### 3.2.2. Significant Academic Pressure Group

(1) Within-group improvement: As presented in Table 3, following the intervention (T2), the significant academic pressure intervention group exhibited significantly lower scores compared to pre-intervention (T1) on the SCL-90 total score and on five dimensions: somatization, obsessive-compulsive, depression, and anxiety (all *p* ≤ 0.026). Effect sizes were all in the large range. Improvements in interpersonal sensitivity, paranoid ideation, and other dimensions did not reach statistical significance after B-H correction (*p* > 0.026). Zero-order correlations (r) between pre- and post-test scores ranged from −0.414 to 0.953. Notably, 8 out of 10 dimensions showed strong positive correlations (r > 0.7), with a correlation of r = 0.787 for the Total Score, indicating high consistency in improvement patterns for the vast majority of dimensions.

(2) Net intervention effect: As shown in Table 4, compared to the significant academic pressure control group, the intervention group demonstrated significantly greater improvements (change scores) on the SCL-90 total score and on four dimensions: obsessive-compulsive, depression, and anxiety (all *p* ≤ 0.026). The effect sizes for these were all large. The net intervention effects for the somatization dimension and other dimensions did not reach statistical significance after B-H correction (*p* > 0.026).

(3) Summary (Significant Academic Pressure Group): The results indicate that for university students with psychological subhealth experiencing significant academic pressure, the group intervention significantly improved their overall psychological health and produced practically meaningful net improvements on three core symptom dimensions: obsessive-compulsive, depression, and anxiety (meeting the criteria of both significant within-group improvement with *p* ≤ 0.026 and notable effect size, and significant net intervention effect with *p* ≤ 0.026 and notable effect size).

**Table 3:** The *t*-test results of paired samples before and after the “significant academic stress group” experimental group.

SCL-90 Dimension	M ± SD		t	*p*	Cohen’s *d*
	Experimental Group Pre-Test	Experimental Group Post-Test			
Somatization	1.595 ± 0.255	1.174 ± 0.208	3.412	0.008	1.079
Obsessive-Compulsive	2.550 ± 0.655	1.600 ± 0.380	7.07	0.000	2.236
Interpersonal Sensitivity	2.511 ± 0.515	2.386 ± 0.447	2.446	0.037	0.773
Depression	2.430 ± 0.721	1.504 ± 0.335	4.307	0.002	1.362
Anxiety	2.200 ± 0.704	1.360 ± 0.357	5.296	0.000	1.675
Hostility	2.108 ± 0.431	2.095 ± 0.479	0.274	0.790	0.087
Phobic Anxiety	2.208 ± 0.557	2.014 ± 0.546	1.927	0.086	0.609
Paranoid Ideation	2.245 ± 0.526	1.980 ± 0.422	2.449	0.037	0.774
Psychoticism	1.300 ± 0.156	1.280 ± 0.148	0.612	0.555	0.194
Total Score	191.5 ± 23.927	115.4 ± 16.447	16.099	0.000	5.091

**Table 4:** The independent sample *t*-test results of the change values between the experimental group and the control group in the “significant academic pressure group”.

SCL-90 Dimension	M ± SD		t	*p*	Cohen’s *d*
	Experimental Group Change Scores	Control Group Change Scores			
Somatization	−0.421 ± 0.390	−0.081 ± 0.32	−2.131	0.047	0.953
Obsessive-Compulsive	−0.950 ± 0.425	−0.380 ± 0.605	−2.438	0.025	1.090
Interpersonal Sensitivity	−0.125 ± 0.162	−0.075 ± 0.221	−0.578	0.570	0.258
Depression	−0.926 ± 0.680	−0.158 ± 0.376	−3.125	0.006	1.398
Anxiety	−0.840 ± 0.502	0.040 ± 0.606	−3.538	0.002	1.582
Hostility	−0.013 ± 0.150	−0.204 ± 0.697	0.848	0.408	0.379
Phobic Anxiety	−0.194 ± 0.318	−0.285 ± 0.583	0.433	0.670	0.194
Paranoid Ideation	−0.265 ± 0.342	−0.234 ± 0.545	−0.152	0.881	0.068
Psychoticism	−0.020 ± 0.103	0.000 ± 0.094	−0.452	0.656	0.202
Total Score	−76.1 ± 14.948	−16.12 ± 3.426	−6.828	0.000	3.054

#### 3.2.3. Family Dysfunction Group

(1) Within-group improvement: As detailed in Table 5, after the intervention (T2), the family dysfunction intervention group showed significantly lower scores compared to pre-intervention (T1) on the SCL-90 total score and on six dimensions: somatization, interpersonal sensitivity, depression, anxiety, hostility, and paranoid ideation (all *p* ≤ 0.026). Effect sizes indicated large effects. Improvements on the obsessive-compulsive, phobic anxiety, and psychoticism dimensions did not reach statistical significance after B-H correction (*p* > 0.026). For pre-post comparisons, zero-order correlations (r) spanned from −0.060 to 0.840 (Total Score: r = 0.840). Most dimensions (7 out of 10) demonstrated moderate to strong positive correlations (r > 0.58), suggesting a stable association between baseline and post-intervention states across core symptoms.

(2) Net intervention effect: As shown in Table 6, compared to the family dysfunction control group, the intervention group demonstrated significantly greater improvements (change scores) on the SCL-90 total score, interpersonal sensitivity, depression, hostility, paranoid ideation, and the anxiety dimension (all *p* ≤ 0.026). The effect sizes were medium to large. The net intervention effects for other dimensions did not reach statistical significance after B-H correction (*p* > 0.026).

(3) Summary (Family Dysfunction Group): The analysis showed that for university students with psychological subhealth resulting from family dysfunction, the group intervention, while improving their overall psychological health, produced particularly significant and practically meaningful improvements on five symptom dimensions: interpersonal sensitivity, depression, anxiety, hostility, and paranoid ideation (meeting the criteria of both significant within-group improvement with *p* ≤ 0.026 and notable effect size and significant net intervention effect with *p* ≤ 0.026 and notable effect size).

**Table 5:** Paired samples *t*-test results before and after intervention for the “Family Dysfunction” experimental group.

SCL-90 Dimension	M ± SD		t	*p*	Cohen’s *d*
	Experimental Group Pre-Test	Experimental Group Post-Test			
Somatization	1.592 ± 0.265	1.304 ± 0.200	2.665	0.026	0.843
Obsessive-Compulsive	2.580 ± 0.547	2.34 ± 0.540	2.395	0.040	0.757
Interpersonal Sensitivity	2.519 ± 0.487	1.887 ± 0.425	5.804	0.000	1.836
Depression	2.480 ± 0.608	1.507 ± 0.325	7.798	0.000	2.466
Anxiety	2.179 ± 0.676	1.450 ± 0.375	4.214	0.002	1.333
Hostility	2.033 ± 0.464	1.150 ± 0.146	5.746	0.000	1.817
Phobic Anxiety	2.064 ± 0.415	1.861 ± 0.416	2.465	0.036	0.779
Paranoid Ideation	2.219 ± 0.497	1.117 ± 0.177	9.031	0.000	2.856
Psychoticism	1.360 ± 0.158	1.341 ± 0.117	0.462	0.655	0.146
Total Score	192.2 ± 24.444	117 ± 17.493	17.48	0.000	5.528

**Table 6:** Independent sample *t*-test results of change values between the experimental and control groups in the “Family Dysfunction” predicament.

SCL-90 Dimension	M ± SD		t	*p*	Cohen’s *d*
	Experimental Group Change Scores	Control Group Change Scores			
Somatization	−0.288 ± 0.342	−0.111 ± 0.345	−1.154	0.264	0.516
Obsessive-Compulsive	−0.240 ± 0.317	−0.490 ± 0.866	0.857	0.403	0.383
Interpersonal Sensitivity	−0.632 ± 0.344	−0.074 ± 0.228	−4.275	0.000	1.912
Depression	−0.973 ± 0.395	−0.182 ± 0.351	−4.734	0.000	2.117
Anxiety	−0.730 ± 0.548	0.020 ± 0.813	−2.418	0.026	1.081
Hostility	−0.883 ± 0.486	−0.050 ± 0.639	−3.282	0.004	1.468
Phobic Anxiety	−0.203 ± 0.260	−0.252 ± 0.430	0.308	0.762	0.138
Paranoid Ideation	−1.102 ± 0.386	−0.313 ± 0.526	−3.828	0.001	1.712
Psychoticism	−0.019 ± 0.130	−0.010 ± 0.099	−0.174	0.864	0.078
Total Score	−75.2 ± 13.604	−9.7 ± 25.32	−7.206	0.000	3.223

## 4. Discussion

This exploratory study aimed to investigate the overall intervention effects and differential characteristics of a group intervention, integrating cognitive behavioral theory (CBT) and social support theory, on university students with psychological subhealth facing distinct core predicaments (traumatic experiences, significant academic pressure, and family dysfunction). The findings suggest the general potential of this integrated intervention model and, more importantly, highlight significant differences in intervention effects among groups with different core predicaments. These preliminary results provide empirical support for advancing the precision-oriented development of mental health services for university students.

### 4.1. General Effectiveness

Consistent with the first research question, the findings indicate that the group intervention integrating CBT and social support theory improved the overall psychological health (as measured by the SCL-90 total score) of university students with psychological subhealth and effectively alleviated their depressive and anxiety symptoms. All three core predicament intervention subgroups demonstrated significant pre-to-post improvements on the SCL-90 total score and on the anxiety and depression factor scores, with these improvements showing significant net effects and large effect sizes compared to their respective control groups. This general positive intervention effect likely stems from the synergistic action of the integrated cognitive-behavioral methods and social support elements within the intervention protocol. Cognitive-behavioral techniques (e.g., identifying and modifying negative cognitions, learning adaptive coping skills for various situations, relaxation training) provided students with practical tools for managing their emotions and thoughts, directly targeting the core symptoms of anxiety and depression (Bentley et al., 2021; Christ et al., 2020; Worsley et al., 2022). Concurrently, the safe and accepting atmosphere fostered by the group environment, along with understanding, emotional support, and shared experiences from peers, effectively enhanced participants’ sense of social connection and belonging, reduced loneliness, and increased self-efficacy and perceived social resources for coping with stress and negative emotions (Cohen & Wills, 1985; Cobb, 1976; Worsley et al., 2022). These common intervention mechanisms provided a universally applicable support framework for university students from different predicament backgrounds, promoting an overall positive shift in their psychological state.

### 4.2. Differential Intervention Effects

Building on the observed general effectiveness of the group intervention, the core finding of this study lies in the revelation that intervention effects exhibited significant differential characteristics depending on the type of core predicament faced by the university students. Even though all groups showed significant improvements in anxiety and depression, reflecting certain common benefits of the intervention, the variations in improvement across other specific symptom dimensions more prominently highlighted the uniqueness of their core predicaments and the potential for a “precision match” between intervention strategies and these unique characteristics. Crucially, since the intervention protocol was standardized across groups, these observed inter-group differences in effects could be largely attributed to the interaction between different population characteristics (core predicaments) and the intervention elements, rather than to substantive differences in the protocol itself.

Traumatic Experience Group: In addition to significant improvements in dimensions such as anxiety and depression, similar to other groups, participants in this group exhibited particularly prominent improvements in interpersonal sensitivity, phobic anxiety, and psychoticism. This may reflect that the intervention effectively addressed and responded to the core symptom cluster associated with post-traumatic stress. Traumatic experiences often lead to impaired interpersonal trust (manifesting as interpersonal sensitivity) (American Psychiatric Association, 2013; Goldstein et al., 2023), heightened vigilance and avoidance of specific situations (anxiety, phobic anxiety) (American Psychiatric Association, 2013; Reuther et al., 2010), and even cognitive distortions or extreme thinking disconnected from reality (potentially reflected in relatively elevated psychoticism factor scores) (Melegkovits et al., 2025; Fung et al., 2023). The group intervention in this study, by providing a safe and protected interpersonal environment and encouraging the expression and normalization of trauma-related emotions, may have directly contributed to repairing damaged interpersonal trust. The cognitive restructuring component likely precisely assisted participants in identifying and correcting trauma-related negative cognitions such as catastrophizing and stigmatization, while specific interpersonal interaction skills training (e.g., boundary setting) also facilitated the rebuilding of secure interpersonal connections. This high degree of congruence between the intervention content and the characteristics of trauma-related core predicaments may be why this group benefited more in these specific dimensions, supporting the potential of comprehensive interventions for addressing complex trauma responses.

Significant Academic Pressure Group: Beyond the common improvements, the specific enhancements for this group were primarily concentrated in the obsessive-compulsive and anxiety dimensions. Academic pressure is often closely associated with perfectionistic cognitive patterns, catastrophic expectations of failure, and the resultant repetitive thoughts and checking behaviors (obsessive-compulsive symptoms) and persistent tension (anxiety) (Hu et al., 2023; Obando Posada et al., 2023). The stress management techniques (e.g., time management, goal decomposition), relaxation training, and cognitive-behavioral techniques (e.g., identifying and challenging irrational beliefs such as “I must succeed every time” or “one failure means total failure,” and lowering perfectionistic standards) included in this study’s intervention directly targeted these cognitive and behavioral patterns closely linked to academic pressure. The significant improvement in obsessive-compulsive symptoms (net effect d = 1.090) is particularly noteworthy, suggesting that the cognitive restructuring and behavioral coping strategies within the group intervention effectively alleviated common pressure-related obsessive thoughts and behaviors among university students.

Family Dysfunction Group: In addition to common improvements, participants in this group showed significant enhancements in dimensions such as interpersonal sensitivity, anxiety, hostility, and paranoid ideation. These areas of improvement align closely with the nature of this group’s core predicament. Prolonged adverse family interaction patterns can easily lead individuals to develop insecure attachment styles in broader interpersonal relationships, manifesting as mistrust and hypervigilance towards others (interpersonal sensitivity, paranoid ideation), suppressed anger and frustration (hostility), and diffuse anxiety (Brown et al., 2021; Lin et al., 2024; Griffith et al., 2020). The group intervention, by providing an interpersonal “laboratory” environment that differed from their families of origin and was more supportive and constructive, allowed participants to explore and practice new, adaptive communication patterns in a safe environment (e.g., techniques for effectively expressing needs and setting healthy interpersonal boundaries, as covered in the intervention). Through being listened to and empathized with, their accumulated negative emotions (such as anger and the frustration of being misunderstood) were processed. Furthermore, they gradually revised negative expectations of others’ intentions formed due to early adverse interpersonal experiences. These intervention elements precisely responded to the core interpersonal and emotional distress triggered by family dysfunction, thereby fostering significant improvements in these specific dimensions.

These differential results suggest that a “one-size-fits-all” intervention model may not be optimal; instead, making targeted adjustments or matching resources based on students’ core predicaments is likely to yield better intervention outcomes.

### 4.3. Theoretical Implications

Firstly, this study provides new empirical evidence for the integrated application of cognitive behavioral theory and social support theory in interventions for psychological subhealth among university students, further revealing the complexity and context dependency of such integrated intervention effects. Secondly, by classifying participants according to their core predicaments, this research underscores the importance of attending to individual differences and specific stressors when understanding and intervening in psychological subhealth issues. This emphasis may contribute to advancing mental health theories from more universal models towards more refined, nuanced frameworks.

### 4.4. Practical Implications and Future Directions

#### 4.4.1. Practical Implications

The findings offer several implications for optimizing university mental health services:

1. Precision Assessment: Service providers should enhance the initial identification of students’ core predicaments. Understanding primary needs facilitates the precision matching of resources, moving beyond a generic approach.
2. Universal Framework with Targeted Adjustments: The integrated CBT and Social Support Theory framework can serve as a universal foundation due to its general effectiveness. However, based on the differential findings, practitioners should flexibly strengthen specific modules (e.g., trust-building for trauma, perfectionism management for academic pressure) to maximize outcomes for specific groups.
3. Resource Optimization: Universities can develop themed groups targeting specific predicaments. For symptoms that showed less improvement in this short-term intervention, integrated modalities (e.g., combining group work with individual counseling or medical services) should be considered.

The ultimate goal is to construct a dynamic, individualized intervention system that can both leverage existing intervention strengths and actively address remaining challenges, rather than adhering to a “one-shot” solution.

#### 4.4.2. Future Research Directions

As an exploratory endeavor, this study opens several avenues for future research:

- Sample Expansion: Future studies should replicate this research with larger, more diverse samples to test generalizability and estimate effect sizes more precisely.
- Mechanisms of Change: Future research should incorporate qualitative methods (e.g., interviews, process analysis) to elucidate how specific intervention elements facilitate change for students with different predicaments.
- Protocol Refinement: More targeted, modular protocols could be developed and compared. For instance, comparing a standardized integrated protocol against specialized modules (e.g., trauma-focused trust building vs. academic perfectionism restructuring).
- Complex Predicaments: This study focused on individual core predicaments. Future research should explore interventions for students with co-occurring predicaments (e.g., academic pressure combined with family dysfunction) and investigate whether they benefit more from a single, longer integrated program or separate, sequential modules.
- Long-Term Follow-Up: Introducing follow-up assessments (e.g., 6 months post-intervention) is critical to evaluating the durability of effects on students’ long-term adaptation.
- Multi-Source Assessment: To reduce self-report bias, future studies should integrate behavioral observations, physiological indicators, or peer ratings to capture intervention effects more objectively.

Through sustained research efforts, it is hoped that a deeper understanding of the complexities of psychological subhealth among university students can be achieved, leading to the development of more effective, precise, and humanistic mental health service systems.

### 4.5. Limitations

Several limitations of this exploratory study must be acknowledged. First, the sample size was small (*N* = 60, *n* = 10 per subgroup). While this resulted from our strict “dual-threshold” screening to ensure the homogeneity of core predicaments, it limits statistical power. Second, the strict sampling strategy implies that findings may be specific to these carefully selected subgroups and may not fully reflect the broader student population (overfitting risk). Third, regarding statistical analysis, the use of multiple *t*-tests on a small sample carries an inherent risk of Type I errors, although we applied the Benjamini–Hochberg correction and a dual criterion (requiring both within-group and between-group significance) for significance to mitigate this. Future studies with larger samples should employ repeated measures ANOVA. Fourth, social desirability bias was not explicitly measured or controlled for; the supportive group atmosphere itself might have produced a “placebo effect.” Future research should include social desirability scales to control for this potential confound. Finally, this study was conducted in a specific cultural context (Chinese universities); caution should be exercised when generalizing these findings to other cultural settings.

## 5. Conclusions

This empirical study indicates that a group intervention integrating cognitive behavioral theory and social support theory can effectively improve the overall psychological health of university students with psychological subhealth and significantly alleviate their common negative emotional states, particularly depression and anxiety. A more critical contribution is the study’s observation that these intervention effects possess significant differential characteristics: the benefits for university students are closely related to the type of core predicament they face (traumatic experiences, significant academic pressure, or family dysfunction), with different focal points of improvement observed across various SCL-90 symptom dimensions. These findings underscore the importance of precise assessment and classification in mental health services for university students. Furthermore, they provide preliminary empirical evidence for developing and implementing differentiated, personalized intervention strategies tailored to different core predicaments, thereby holding significant theoretical and practical implications for enhancing the targeting and effectiveness of mental health services for this population.

## Data Availability

The data presented in this study are available on request from the corresponding author. The data are not publicly available due to privacy and ethical restrictions.
